# Diurnality is consistently different between individuals and decreases with disease or stressful events in dairy calves

**DOI:** 10.1038/s41598-025-09983-z

**Published:** 2025-07-25

**Authors:** Matthew Thomas, Francesca Occhiuto, Jorge A. Vázquez-Diosdado, Jasmeet Kaler

**Affiliations:** School of Veterinary Medicine and Science, University of Nottingham, Sutton Bonington Campus, Leicestershire, LE12 5RD UK

**Keywords:** Behavioural methods, Bioinformatics

## Abstract

Changes in behavioural rhythms of livestock can be useful indicators of disease or stress before visible signs appear. Using precision livestock technologies, it is possible to measure behavioural patterns and compute the diurnality, determined by proportion of activity that happens during the daytime. Accounting for individual variation in behaviour due to personality or predictability differences is an essential step for the early detection of disease or stress. Here we aimed to detect individual differences and changes in daily rhythms. We use ultra-wideband location sensors to measure the diurnality of 285 calves across the weaning period and two housings. Calves were shown for the first time to have consistent individual differences in diurnality (the repeatability was 0.29 and 0.39 in each housing) and to differ in their predictability (CVp = 0.15 and CVp = 0.28 in each housing). This was the first time that a decrease in diurnality was detected when calves were experiencing disease or the stress of disbudding, as the diurnality index decreased by 0.07 and 0.45 respectively. Diurnality increased with age and decreased during weaning and in the summer months. These results highlight the importance of studying individual variation and daily rhythms of activity for the development of automated disease detection tools.

## Introduction

Maintaining a good standard of health and welfare for farm animals has multiple benefits, including improving production, reducing management cost^1^, and lessening the ethical concerns of consumers^2^. However, detecting negative states in farm animals promptly and reliably is often difficult. As most livestock species are prey animals, they tend to hide subtle signs of illness^3^, meaning that visible clinical symptoms may only be identified when the disease is more severe. Subtle changes in behaviour measured using precision livestock technologies have been used successfully to detect disease in cattle^4–8^, but very few studies have focused on changes in the patterns of daily activity^9,10^.

Many physiological and behavioural expressions in animals follow periodic daily patterns governed by the circadian rhythm^11^. For example, the body temperature of mammals follows predictable changes throughout the day, which in cattle is evident from about 30 days old^12,13^, and the activity pattern of most animals also follows a rhythm which is synchronised with the day^11^. Some species, including cattle, perform most of their movement during daylight hours, making them diurnal^13^. Both adult cows and calves appear to have two peaks in daily locomotor activity, one in the morning and one in the afternoon, and very little activity is performed during the night^9,14,15^. Changes in the daily activity patterns have been associated with disease or stress in adult cows^9,10^, but very few studies have tested this in calves^15,16^. In addition, artificial lighting and conditions imposed by farming on animals may disrupt their natural rhythm, which has been shown to cause health issues in humans but not tested in farm animals^17^. Therefore, understanding the daily rhythms of farm animals is of great importance.

Individual variation in behaviour within a population has been observed in many species through the measure of repeatability, which is the proportion of the total variation that is due to individual differences^18^. Calves have been proven to be consistently different from one another in their total daily movement^19,20^ and feeding behaviours^21,22^, social network position^23^ and in a variety of personality tests^24,25^. Individual variation in the daily rhythm of activity of calves was also described^16^, but the study is constrained by small sample size and short duration and no previous study has ever measured the repeatability of daily rhythms. Individuals also vary from one another in their level of predictability, which is the residual intra-individual variation (rIIV) that is not explained by individual identity or environmental factors^26,27^. This type of variation was also demonstrated in the total daily movement and feeding of calves^19,21,22^, but was never computed for daily rhythms of any animal. Quantifying individual differences in behaviour is a fundamental step if we intend to use behavioural changes as indicators of negative outcomes in farm animals but it is often overlooked.

Measuring behavioural rhythms and individual differences requires very granular data and robust analysis relies on large sample sizes, the combination of which results in datasets that are too time consuming to collect by observation. However, the use of precision livestock technologies, which are becoming increasingly widespread on farms^28–30^, allows the automated gathering of such data for the first time. In this study we aim to quantify the behavioural rhythms of calves, how they vary between individuals and how they are affected by environmental effects or disruptive events. We used neck mounted location sensors to measure activity levels. We then used the activity to compute the flowing two measures: the diurnality index (DI), which measures the proportion of activity occurring during daytime, and the active phase duration, which measures the daily duration of high activity. We then quantified the variation in DI between individuals (repeatability) and within individuals (predictability) from birth and throughout the weaning process. We used these data to test the following predictions for the first time: as cattle are normally diurnal, we predicted that calves would have a positive DI (prediction 1) and that the DI would increase with age due to the development of the circadian clock in the first few weeks of life (prediction 2). We also predicted that calves would have a higher active phase duration in the summer months due to increased daylight hours (prediction 3). Finally, we predicted that calves would show individual variation between individuals (personality) and within individuals (predictability), as personality and predictability differences have been observed in other aspects of calf behaviour (prediction 4) and that any disease or stressful events, such as disbudding, would cause a decrease in DI due to a disruption in the daily rhythm (prediction 5).

## Materials and methods

This study was carried out at the Centre for Dairy Science Innovation at the University of Nottingham, UK (latitude 52.83871, longitude − 1.24972) between April 2021 and December 2023 and was approved by the Ethics Committee of the School of Veterinary Medicine and Science, University of Nottingham (unique reference number 1481150603). This study was performed in accordance with relevant guidelines and regulations of the School of Veterinary Medicine and Science, University of Nottingham and all methods are reported in accordance with ARRIVE guidelines^31^.

In total 285 Holstein-Friesian heifer calves were included in the analysis from 19 cohorts of between 10 and 16 calves per cohort. No a priori sample size calculation was conducted for this study. This study used available data from a previously conducted experiment, which determined the sample size. From birth, calves were housed in pairs in straw bedded pens measuring 3 m x 2 m. They were disbudded when they reached a minimum of 14 days of age as per farm protocols by trained farm staff using heated iron and under local anaesthetic. Calves were moved to a group pen of 6 m x 10 m when enough pairs had reached a minimum of 21 days old. The mean age for calves in the group housing was 76.3 days with sd 25.6 days. In both housings calves were fed from a computer-controlled milk feeding machine, which recognised the identity of the calf via RFID ear tags and allocated the correct amount of milk replacer based on the age. The machine automatically allocated 2 L of milk replacer every 2 h from 05:00AM up to a total of 6 L at 2 days of age, increasing gradually with age and reaching 10 L per day at 40 days old. After moving to the group housing all calves where they were fed 10 L of milk replacer from the same type of feeding machine. The gradual weaning process started for all the calves after 38 days in the group housing, when the milk allowance decreased gradually for 20 days until the calves were fully weaned. Both housings had troughs containing concentrates, chopped straw and water, which the calves had ad-lib access to.

Location data was collected using collars with Ultra-Wideband Sewio Leonardo iMU tags, (Noldus, Wageningen, the Netherlands), which reported the coordinates of each calf with frequency of 1 Hz, in accordance with Occhiuto, Vázquez-Diosdado^19^. Any days when at least 15 min of data were missing were excluded from the analysis, in addition, calves without a recorded disbudding date (119) and less than 15 valid days in each housing (84) were excluded. In total data was available for 77 calves from 8 cohorts totalling 1,292 calf-days in the pair housing and for 280 calves from 19 cohorts totalling 17,198 calf-days in the group housing. Calves in the group housing pens were health scored twice a week by trained researchers according to the Wisconsin-Madison scoring system^32^. Health scores were imputed using an exponential moving average with a window size of 4, values were imputed for the Wisconsin score, temperature score and cough score.

Diurnality index (DI) is calculated as a ratio of activity, in the prescribed “day-time” vs. the prescribed “night-time”. In this study sunset and sunrise data for each year was obtained from the National Oceanic and Atmospheric Administration (NOAA), and for each year the average sunrise and sunset times were calculated (Table 1). Day-time was defined between this average sunrise and average sunset, and night-time was defined between sunset times and sunrise. The calculation of diurnality (D) is as follows where $\:{C}_{d\:}$is the sum of distance travelled in the defined day-time and $\:{C}_{n}$is the sum of distance travelled in the defined night-time^33^.

$$\:D=({C}_{d}-{C}_{n})/({C}_{d}+{C}_{n})$$

The result of this equation means values of diurnality (D) can range from 1, where all activity occurs in the day-time only, and − 1, where activity occurs only in the night-time. Values between 1 and − 1 represent the degree to which activity is favoured in one time over another. Where the majority of the activity occurs in the day-time, D is positive and an animal is said to be diurnal, where D is negative indicating that the majority of activity occurs in the night-time, an animal is said to be nocturnal.

**Table 1:** Average sunset and sunrise times for each year of data included in the study. Sunset and sunrise times as obtained from the National oceanic and Atmopsheric administration (NOAA: https://gml.noaa.gov/grad/solcalc/) for the centre for dairy science innovation at the university of Nottingham UK (latitude 52.83871, longitude − 1.24972).

Year	Av. Sunrise	Av. Sunset
2021	06:32:02	18:49:14
2022	06:32:03	18:49:15
2023	06:31:46	18:49:45

**Fig. 1:**
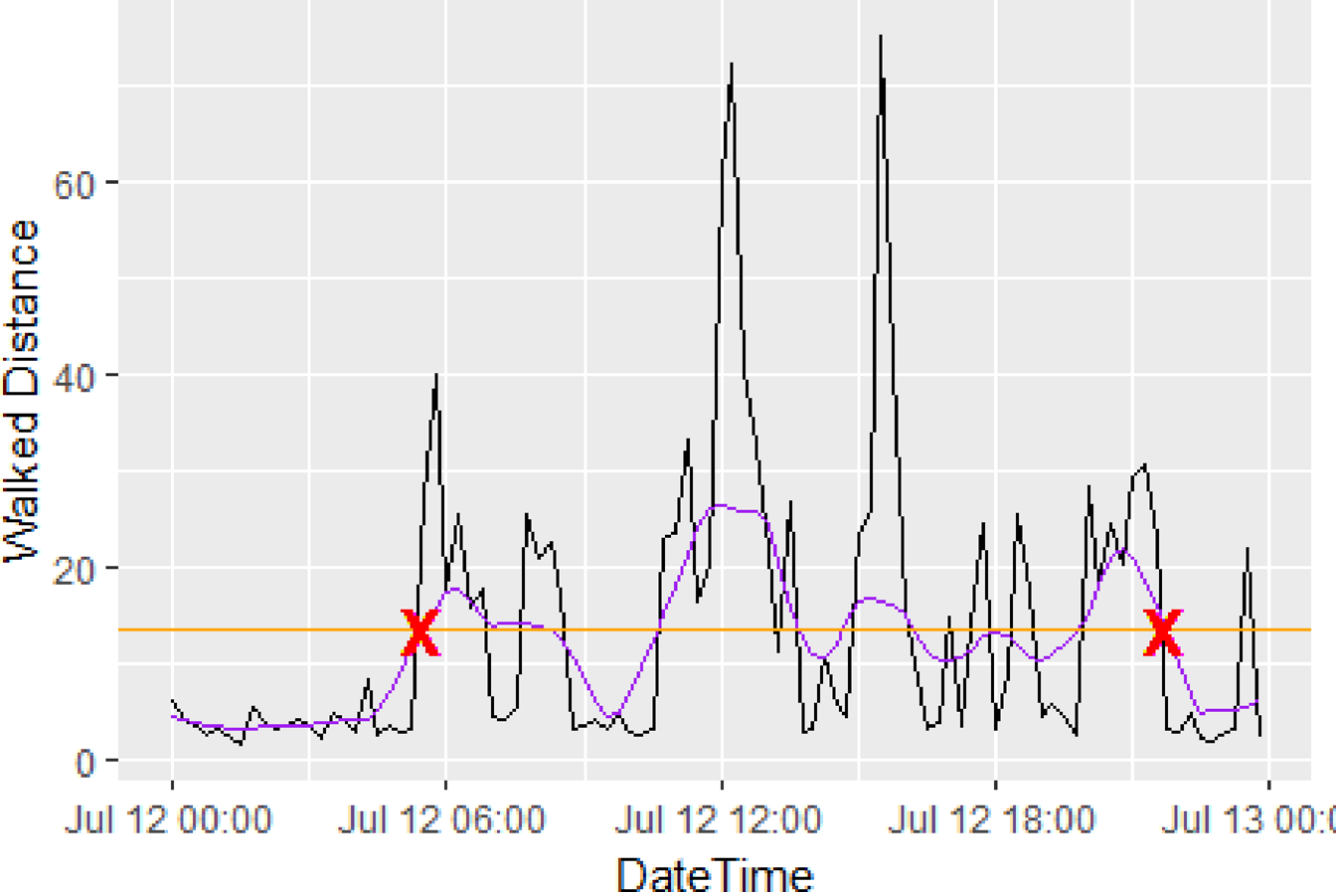
Raw distance travelled (m) by the calf where there is one point per 15 min interval (black line), mean distance travelled (m) by the calf on that day (orange line), smoothed distance travelled (m) by the calf (purple line) and the onset and offset times (red crosses).

To calculate activity onset and offset times a smoothed line was fitted using the LOWESS algorithm^34^ (locally weighted polynomial regression) with span 1/8 and the activity onset time of a calf for a given day was defined as the earliest time (for onset) and last time (for offset) in the day that their smoothed activity was recorded above the mean for that day. The active phase duration for each calf for each day was defined as the difference in time between the activity onset time and the activity offset time. Figure 1 illustrates how the onset and offset time were calculated for an example calf on a single day.

To test the effects of different variables on diurnality and quantify the individual variation two separate double hierarchical generalised linear models (DHGLMs) were run, one for each housing, herein referred to as the pair housing model and group housing model respectively, using the “brms” package in R 4.3.2^34,35^.

DHGLMs allow for the variation between calves (repeatability) and variation within individual calves (predictability) to modeled separately. Models were ran with non-informative priors, for 5000 iterations with a warmup of 2000. Four chains were used with a thinning of 4 and all chains were converged with Rhat < 1.05.

In both models diurnality was the outcome variable, calf ID and cohort number were included as random effects and age in months and month of the year as fixed effects. In the pair housing model an additional categorical variable “disbudding” was added as a fixed effects and set as “true” in the day following the disbudding event. In the group housing two additional variables were added to measure the effects of the weaning (“pre-wean”: mean 37.4 days, “weaning”: mean 38.8 days or “weaned”: mean 22.2 days) and the health status based on the imputed values from the health scoring. A calf was considered sick on a given day if the imputed total health score was greater than 4.5, the rectal temperature was greater than 39.4 or the cough score was greater than 2. In total, 2336 calf-days were labelled as sick and 14,862 were labelled as healthy, 221 calves had at least one sick day. Due to a lack of data covering March and November in the pair housing, Month was fitted as a polynomial with order 2 in order to allow a cyclical fit over the year.

## Results

### Diurnality

**Fig. 2:**
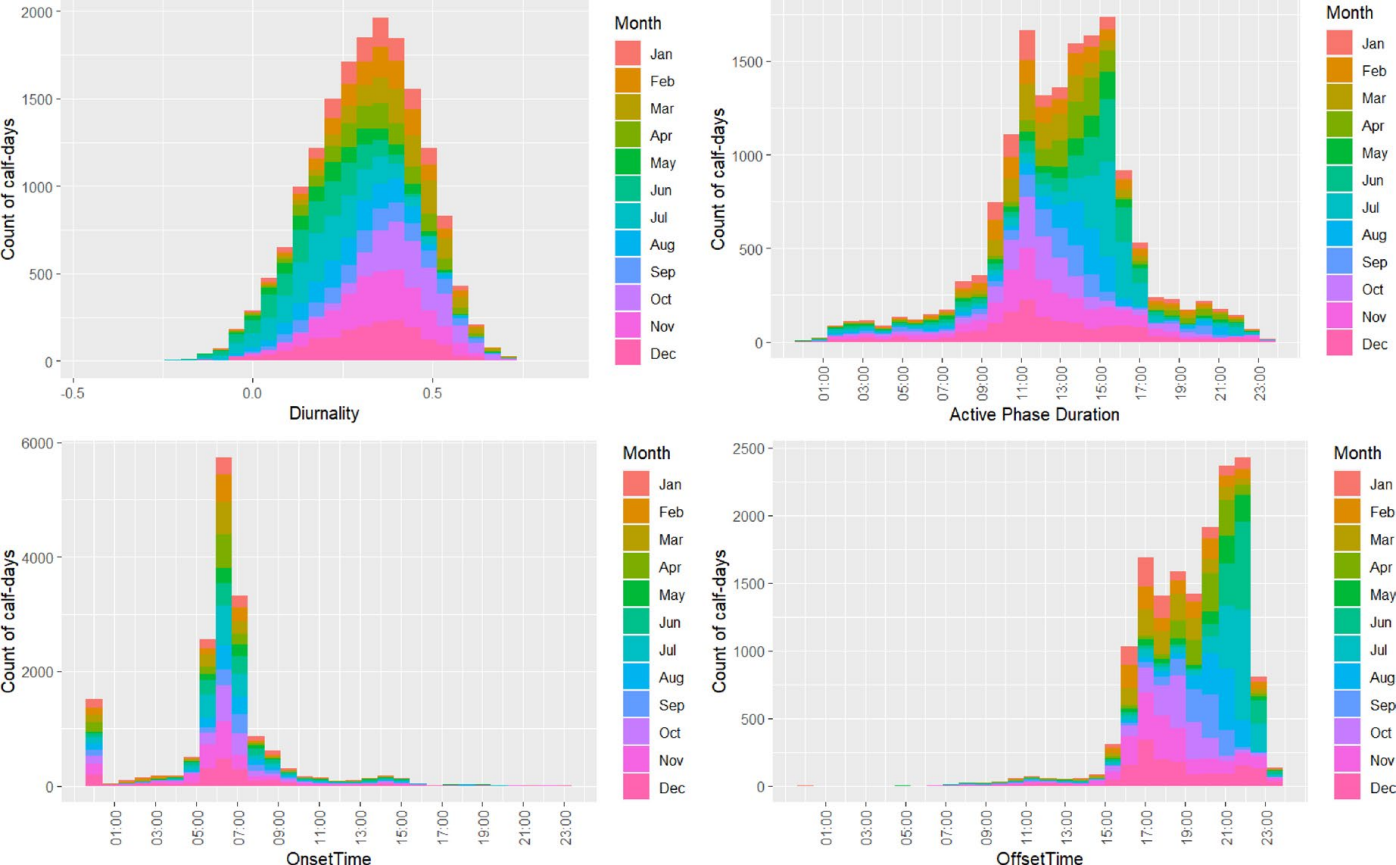
(**a**) Stacked histogram showing the distribution of daily diurnality values for 17,198 calf-days from 280 group housed dairy calves in 19 cohorts coloured by month (Jan – Dec). The y-axis indicates the number of calf-days for each diurnality value on the x-axis. (**b**) Stacked histogram showing the distribution of active phase duration for 17,174 calf-days from 280 group housed dairy calves in 19 cohorts coloured by month (Jan – Dec). The y-axis indicates the number of calf-days for each time value on the x-axis. (**c**) Stacked histogram showing the distribution of onset times for 17,174 calf-days from 280 group housed dairy calves in 19 cohorts coloured by month (Jan – Dec). The y-axis indicates the number of calf-days for each time value on the x-axis. (**d**) Stacked histogram showing the distribution of offset times for 17,174 calf-days from 280 group housed dairy calves in 19 cohorts coloured by month (Jan – Dec). The y-axis indicates the number of calf-days for each time value on the x-axis.

**Fig. 3:**
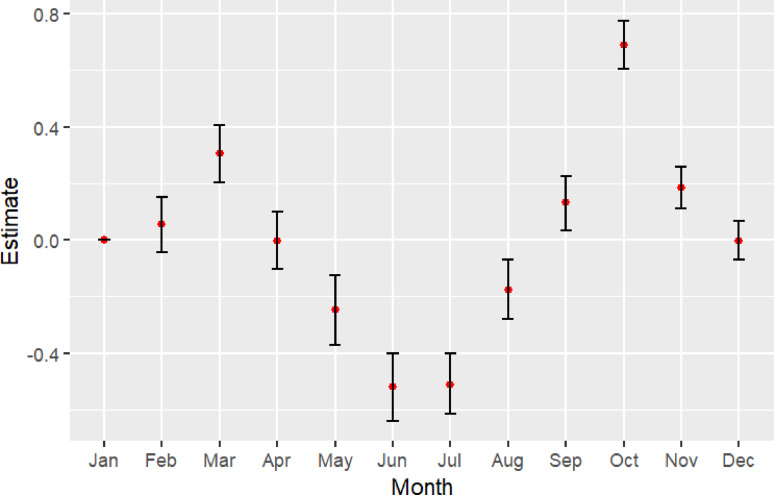
Estimate of the effect of month on diurnality for calves in the group housing. Red points indicate the estimate flanked by capped black bars showing the range of the 95% CI. (Calves = 280, cohorts = 19, calf-days = 17,198)

Across both housings calves were broadly diurnal (Fig. 2a) in their locomotive behaviour and favoured daylight hours for their locomotive behaviour (DI = 0.31, SD = 0.15), however as diurnality is not equal to 1, this shows that calves performed some locomotion in the night-time. Diurnality tends to be slightly higher in the winter months than the summer months (Fig. 3).

### Onset, offset and active phase duration

The average onset time of activity for the calves was 6:15AM (SD = 2.87 h) and the average offset time was 19:21PM (SD = 2.42 h). Onset times appeared to be tightly clustered around 06:00AM except for a small peak at midnight, which would occur from calves who were night-active (Fig. 2c). Offset times were considerably more spread-out, and calves tended to be active later in the summer months and earlier in the winter months (Fig. 2d). The average active phase duration was 13:05 h (SD = 3.58 h) but tended to be longer in the summer months and shorter in the winter months, which is driven largely by the later offset times in summer (Fig. 2b).

### Group housing model

**Fig. 4:**
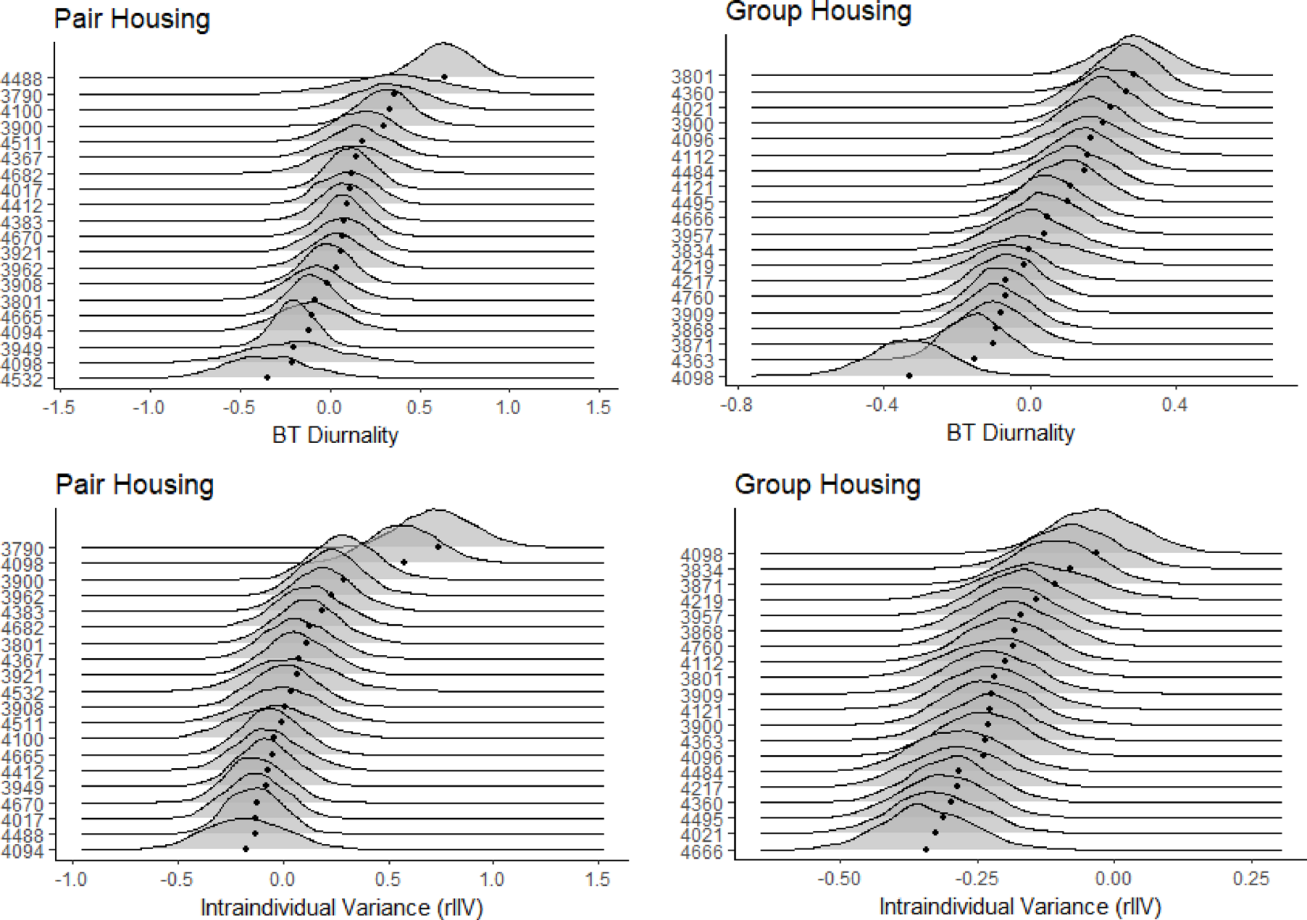
(**a**) Posterior distributions of the behavioural type (BT) for diurnality in the pair housing on a sample of 20 calves. The black dots represent the mean for each calf and the ridges indicate the posterior 95% credible interval. (**b**) Posterior distributions of the behavioural type (BT) for diurnality in the group housing on a sample of 20 calves. The black dots represent the mean for each calf and the ridges indicate the posterior 95% credible interval. (**c**) Posterior distributions of the residual intraindividual variation (rIIV) of diurnality in the pair housing on a sample of 20 calves. The black dots represent the mean for each calf and the ridges indicate the posterior 95% credible interval. (**d**) Posterior distributions of the residual intraindividual variation (rIIV) of diurnality in the group housing on a sample of 20 calves. The black dots represent the mean for each calf and the ridges indicate the posterior 95% credible interval.

The diurnality of calves increased with age (0.17; 95% CI: 0.13–0.20), decreased as they were going through the gradual weaning (-0.12; 95% CI: -0.17, -0.08) and after they were fully weaned (-0.25; 95% CI: -0.32, -0.18) (Table 2). Diurnality was lowest in the summer months of June (-0.52; 95% CI: -0.64, -0.40) and July (-0.52; 95% CI: -0.64, -0.40) and highest in the spring and autumn months of March (0.31; 95% CI: 0.20, 0.41) and October (0.69; 95% CI: 0.61, 0.78) (Fig. 3). Calves diurnality also decreased when they were sick (-0.07; 95% CI: -0.11, -0.03). Diurnality was repeatable in the group housing indicating consistent individual differences (*R* = 0.29; 95% CI: 0.16, 0.42) (Fig. 4b), and there were consistent individual differences in predictability between calves (CVP = 0.15; 95% CI: 0.14, 0.17) (Fig. 4d).

**Table 2:** Effect estimates on diurnality for the fit of the group housing model with estimates for the repeatability and predictability. (Calves = 280, cohorts = 19, calf-days = 17,198).

Effect		Estimate	95% CI
Age_Months		0.17	[0.13, 0.20]
Wean Status	Pre-wean	Reference	
	Weaning	-0.12	[-0.17, -0.08]
	Weaned	-0.25	[-0.32, -0.18]
Month	Jan	Reference	
	Feb	0.05	[-0.04, 0.15]
	Mar	0.31	[0.20, 0.41]
	Apr	0.00	[-0.10, 0.10]
	May	-0.25	[-0.37, -0.13]
	Jun	-0.52	[-0.64, -0.40]
	Jul	-0.51	[-0.62, -0.40]
	Aug	-0.18	[-0.28, -0.07]
	Sep	0.13	[0.03, 0.23]
	Oct	0.69	[0.61, 0.78]
	Nov	0.18	[0.11, 0.26]
	Dec	0.00	[-0.07, 0.07]
Health Score	Healthy	Reference	
	Sick	-0.07	[-0.11, -0.03]
Repeatability		0.29	[0.16, 0.42]
Coefficient of variation in predictability		0.15	[0.14, 0.17]

### Pair housing model

Similarly to the group housing, between individual effects show calves increased in their diurnality as they aged (0.62; 95% CI: 0.35, 0.91) (Table 3). A similar seasonal pattern was also observed. In the day following disbudding diurnality was significantly lower (-0.45; 95% CI: -0.72, -0.20). Diurnality was also repeatable in the pair housing indicating consistent individual differences (Fig. 4a) (*R* = 0.39; 95% CI: 0.20, 0.62), and there were consistent individual differences in predictability between calves (Fig. 4c) (CVP = 0.28; 95% CI: 0.24, 0.32).

**Table 3:** Effect estimates on diurnality for the fit of the pair housing model with estimates for the repeatability and predictability. (Calves = 77, cohorts = 8, calf-days = 1,292).

Effect		Estimate	95% CI
Age	Age_Months	0.62	[0.35, 0.91]
Month	Poly 1	-14.27	[-35.55, 1.72]
	Poly 2	8.76	[0.62, 18.80]
Disbudding	False	Ref	
	True	-0.45	[-0.72, -0.20]
Repeatability		0.39	[0.20, 0.62]
Coefficient of variation in predictability		0.28	[0.24, 0.32]

## Discussion

This study measured calf diurnality and the is the first to associate diurnality with age, weaning, disease and stress events, as well as the first to quantify the repeatability and predictability of diurnality in animals. The use of precision livestock technologies enabled the collection of 13,906 calf-days of activity data with high granularity for 285 calves. The length and breadth of data in this study goes further than any other study within the literature to show for the first time detailed estimates of the degree of diurnality as well as it’s variation within and between calves, the seasonal pattern and the effect of illness and stressful events. Linking diurnality with these outcomes could be useful to improve the current methods for early detection of adverse events in farm animals, while also adding to the evidence of individual differences in activity patterns.

Generally, adult cattle are known to focus their locomotor activity during the day^36,37^. The only previous study that quantified calf diurnality did so in weaned calves raised at pasture and also found the activity to more prevalent during daytime hours^38^. Our results extend this finding to pre-weaned dairy calves in an indoor-housing system. In addition, the fact that our study spanned across 33 months, enabled us to measure the effect of season and to demonstrate that diurnality is lower in the summer months. As we choose a fixed day length year-round, this could be reflecting that calves are active for longer due to longer days. However, the onset of activity remains consistent, while the offset is generally later in the summer, indicating that the appearance of daylight is not the main trigger of activity, and the release of a fresh allocation of milk replacer may be encouraging the calves to start moving.

In both housings diurnality increased with age, with a more marked increase in the pair housing. An analysis of the body temperature rhythmicity in calves from birth established that the circadian rhythm is not apparent at birth but develops within two months^12^. This is consistent with our findings as with a stronger circadian rhythm being developed, calves are likely to settle in a pattern of diurnal activity and therefore we can conclude that daily activity patterns also develop in the first months of life. Despite this trend with age, diurnality decreases during the gradual weaning period and after the weaning is complete. The change from the automatic milk feeder to ad-lib solid food requires calves to spend longer eating and ruminating to consume the necessary calories as well as allowing them to spread out their meals throughout the day without any restrictions of the automatic feeder. These factors would contribute to the lower diurnality seen in this study but a comparison with ad-lib milk feeding would help discern these effects.

The occurrence of respiratory disease and the day after the disbudding event are both associated with a decrease in diurnality, indicating that this measure could be used to detect health and welfare issues. Disease in calves has been shown to be associated with changes in behaviour, including changes in lying behaviour^39–41^, reduced walked distance, slower feeding, shorter meals^4^ and fewer unrewarded visits^42^ as well as changes in the social network position^43^. When calves are sick, or recovering from a stressful event, it is likely that their natural rhythm of activity will be disrupted, with more rest breaks throughout the day, resulting in a more even distribution of movement in the 24 h and lower diurnality rather than activity concentrated in the daylight hours. Previous studies in adult cows were able to demonstrate a disruption to the daily activity rhythms when cows are ill or experiencing relocation stress^9,10^. Although this had not been tested directly in calves previously, Veissier, Le Neindre^15^ monitored the circadian rhythm of activity of 40 heifers and found a disruption that coincided with an outbreak of pneumonia, which would support our findings.

Our result that diurnality is repeatable adds to the existing evidence of consistent individual differences between calves in several behaviours^19–21,24,25,44^, but this is the first time that a daily pattern and not a static measure of behaviour is assessed in this way. This result means that the proportion of activity that a calf performs during the daytime depends on the identity of the calf itself, indicating that it may be driven by personality^45,46^. The fact that the feeding pattern was constrained by the automatic feeder for the majority of the trial may have encouraged all the calves to start their daily activity at the time that the daily allocation was released. Therefore, had the calves been fed *ad-libitum*, the individual differences may have been even more pronounced. There is growing support for the definition of animal personality to include any consistent differences in behaviour between individuals^19,47,48^ and our results show that diurnality fits this definition and may be considered a personality trait. Differences in diurnality may be linked to differences in foraging strategy or dominance hierarchy, as individuals may choose to perform their activities at night to avoid competition or confrontation with others. In addition, if we aim to use diurnality or other measures of daily activity patterns to detect disease or stress, it is essential to consider the individual baseline to achieve more effective detection tools, as has been done for existing detection tools^49^.

Calves were also different in their levels of predictability, meaning that some individuals were more consistent than others in their diurnality throughout the study. Outside of human personality research, predictability is very rarely measured in animals^19,22,50,51^. Variation in the level of predictability may reflect different strategies present in the population. While higher predictability may benefit animals when the daily routine is consistent, more unpredictable individuals, who rely on opportunistic behaviour, may be more adaptable and resilient to change, but evidence of any associations to fitness is still lacking. In this study the predictability difference between calves was higher in the pair housing compared to the group. This indicates that in the pair housing, when calves are younger, some may already be settled into a pattern of daily activity, while others do so later in life once they are moved to the group housing. However, in this case we cannot distinguish between the effects of housing and age and therefore further testing of individual differences in the onset of circadian rhythms are needed to confirm this.

Overall, these results highlight the potential of using precision livestock technologies to measure daily patterns of behaviour in detail, for extended periods of time. When accounting for individual variation, diurnality has proven to be a potentially useful measure of calf behaviour, which is linked with health and welfare. This study demonstrated that the diurnality index of a calf is consistently different from others in the population from a very young age and is negatively affected by disease and stress. Using this measure as a detection tool for such events would greatly improve the individualised and timely detection of issues on farms, which would otherwise require time-consuming and subjective observations, and ultimately lead to better animal welfare.

## Data Availability

The datasets generated during and/or analysed during the current study are available from the corresponding author on reasonable request.
